# Nanoparticle-Based Chemotherapy Formulations for Head and Neck Cancer: A Systematic Review and Perspectives

**DOI:** 10.3390/nano10101938

**Published:** 2020-09-29

**Authors:** Jefferson Muniz de Lima, Paulo Rogerio Bonan, Danyel Elias da Cruz Perez, Michael Hier, Moulay A. Alaoui-Jamali, Sabrina Daniela da Silva

**Affiliations:** 1 Department of Otolaryngology Head and Neck Surgery, Sir Mortimer B. Davis-Jewish General Hospital, McGill University, Montreal, QC H3T 1E2, Canada; jefferson.idalino@gmail.com (J.M.d.L.); mhier@jgh.mcgill.ca (M.H.); 2 Segal Cancer Centre and Lady Davis Institute for Medical Research, Sir Mortimer B. Davis-Jewish General Hospital, Departments of Medicine, Oncology, and Pharmacology and Therapeutics, Faculty of Medicine, McGill University, Montreal, QC H3T 1E2, Canada; moulay.alaoui-jamali@mcgill.ca; 3 Department of Clinical and Social Dentistry, Faculty of Dentistry, Federal University of Paraiba (UFPB), Joao Pessoa 58051-900, PB, Brazil; pbonan@yahoo.com; 4 Department of Clinical and Preventive Dentistry, School of Dentistry, Universidade Federal de Pernambuco, Recife 50740-521, PE, Brazil; danyel.perez@ufpe.br

**Keywords:** nanoparticles, targeted therapeutics, head and neck cancer, selective drug delivery

## Abstract

Head and neck cancer (HNC) is a complex and heterogeneous disease associated with high mortality and morbidity worldwide. Standard therapeutic management of advanced HNC, which is based on radiotherapy often combined with chemotherapy, has been hampered by severe long-term side effects. To overcome these side effects, tumor-selective nanoparticles have been exploited as a potential drug delivery system to improve HNC therapy. A combination of MEDLINE, EMBASE, Cochrane Oral Health Group’s Trials Register, Cochrane Central Register of Controlled Trials (CENTRAL) and ClinicalTrials.gov from inception up to June 2020 was used for this systematic review. A total of 1747 published manuscripts were reviewed and nine relevant references were retrieved for analysis, while eight of them were eligible for meta-analysis. Based on these studies, the level of evidence about the efficacy of nanoformulation for HNC therapy on tumor response and adverse side effects (SAE) was low. Even though basic research studies have revealed a greater promise of nanomaterial to improve the outcome of cancer therapy, none of them were translated into clinical benefits for HNC patients. This systematic review summarized and discussed the recent progress in the development of targeted nanoparticle approaches for HNC management, and open-up new avenues for future perspectives.

## 1. Introduction

Head and neck cancer (HNC) is a complex multifactorial disease that originates in the epithelial layer of mucosa of the upper aerodigestive tract, including the oral cavity, pharynx and larynx showing microscopic evidence of squamous differentiation [1,2]. The main risk factors for HNC are tobacco smoke and alcohol consumption, as well as human papilloma virus (HPV) infection. Therapeutic decisions for patients with HNC are primarily based on clinical and pathological tumor stage [1]. It is estimated that 60% of the patients are diagnosed with advanced disease (stage III and IV) leading to low survival rates. In these cases, the treatment consists of surgical ablation followed by adjuvant radiation or chemoradiation (CRT) [3,4]. Despite recent advances in these therapeutic modalities, 50–60% of the patients develop regional relapses or distant metastasis within two years [5]. Patients with recurrent and/or metastatic disease have a median survival lower than 12 months [6], in part due to the limitations of conventional treatments, in particular the severe side effects that worsen quality of life [7].

Nanotechnology based therapy approaches have attracted great interest in oncology in recent years. Nanoformulations, a class of multifunctional materials with diameters of 1-100nm, can act as carriers for drugs and targeting ligands to optimized cancer therapy. The United States Food and Drug Administration (FDA) categorized nanomaterials based on the delivery vehicle or carrier as liposomal, polymeric, albumin-bounds, polymer-bounds, and inorganic particles [8]. These materials have been explored to overcome the biological barriers to cancer treatment due to their unique features such as a large surface area allowing conjugation to biologically active molecules, structural properties (optical, electronic, catalytic and magnetic) and a long time circulation in blood compared with small molecules. Furthermore, a plethora of nanomaterials has been developed to load sufficient drugs and accurately delivery to the tumor site with excellent biocompatibility, biodistribution and biodegradation resulting in lower systemic toxicity [9]. In HNC, early studies in nanotechnology have been designed to overcome the lack of the specificity of conventional chemotherapeutic agents to target cancer cells [10]. In this systematic review, we summarized and discussed the recent progress in the development of targeted nanoparticles systems for HNC therapy opening new avenues for future opportunities of investigations in the field.

## 2. Materials and Methods

The systematic review and meta-analysis were performed in accordance with the protocol of interventions for the treatment of oral and oropharyngeal cancers statement and Cochrane Handbook for Systematic Reviews of Intervention. The tested hypothesis was to establish if the nanoformulation of chemotherapy drugs is able to improve HNC treatment response and prevent side effects. This study did not require ethical approval or informed consent, as the analyses were carried out based on data from previously published clinical trials.

### 2.1. Literature Search

A systematic literature search was conducted in MEDLINE (1946 to present), EMBASE via OVID (1980 to present), Cochrane Oral Health Group’s Trials Register, Cochrane Central Register of Controlled Trials (CENTRAL) (The Cochrane Library, current issue) and ClinicalTrials.gov from inception to June 10, 2020. In addition, World Health Organization (WHO) International Clinical Trials Registry Platform (ICTRP) (http://apps.who.int/trialsearch/), Current Controlled trials (www.controlledtrials.com) and Clinical Trials (www.clinicaltrials.gov) were searched for HNC. The results were compiled using the bibliographic management software EndNote X9 3.2 (Thomson Reuters).

The complete search strategy is listed in the Appendix A in Supplementary Materials. Briefly, the search included patients with squamous cell carcinoma of head and neck according to International Classification of Diseases for Oncology (ICD-O) [11] codes as C00 (lip), C01-C02 (tongue), C03 (gum), C04 (floor of mouth), C05 (palate) and C06 (other unspecified parts of mouth), C09 (tonsil), C10 (oropharynx), C11 (nasopharynx), C12 (pyriform sinus) and C13 (hypopharynx). The outcomes were: “tumor response”, “overall survival”, “disease free survival”, “progression free survival”, “locoregional control”, “recurrence”, “severe adverse effects (SAE)” and “quality of life”.

The list of relevant references was searched, and the authors were contacted in order to identify unpublished or ongoing trials. Any potentially relevant meeting abstracts and articles found in their reference lists were reviewed and considered for inclusion. Two investigators independently reviewed the articles for eligibility. The references retrieved in this study are listed in the EndNote Library in the Supplementary Material S1.

### 2.2. Study Selection: Inclusion and Exclusion Criteria

This research did not retrieve randomized clinical trials because only cohort studies using single-arm clinical trials are currently published. Due to the lack of evidence, only clinical studies at Phases I or II, including more than 50% of patients with HNC, were included. Then, the following criteria were used for inclusion in the meta-analysis: clinical trial where at least one outcome was reported, such as progressive disease (PD), partial response (PR), stable disease (SD), and adverse events (AEs). The exclusion criteria involved non-English papers, single case reports, letters to editor and reviews.

### 2.3. Data Extraction and Study Quality Assessment

Two authors independently extracted data from titles and abstracts. The full report was retrieved when the studies met the inclusion criteria or if the information was not sufficient in the title or abstract to make a clear decision. Articles were graded using the Oxford Centre for Evidence Based Medicine (CEBM) levels of evidence [12]. Discrepancies were identified and resolved through discussion. Missing data were requested directly to the authors via e-mail. Studies rejected at this or subsequent stages were recorded in the characteristics of excluded studies table, and reasons for exclusion noted. The clinical characteristics of the studies are presented in the Table 1. For each clinical trial, the following details were extracted and presented: study characteristics (first author, journal, year of publication, country, multi or single center), trial design characteristics (study design, outcome measurement, therapy regimen), study population (primary tumor location, median age, number of patients evaluated for efficacy and safety endpoints), intervention details (type of intervention, timing, dose, mode of administration and duration, concomitant treatments) efficacy results (PD, PR and SD) and SAE outcomes (Table 2 and Table 3). A standardized, pre-piloted form adapted from the Cochrane Collaboration was used to extract data from the included works. Data Extraction Form.

### 2.4. Risk of Bias Assessment

Once we were not able to find reported randomized clinical trials, all the studies were classified with high-risk of bias according on Cochrane Handbook for Systematic Reviews of Interventions version 5.1.0 [13] These parameters included details of sequence generation, allocation concealment, treatment blinding, completeness of outcomes data, and presence of selective outcome reporting.

### 2.5. Types of Outcome Measures

The primary outcome was the clinical response expressed as tumor volume, as well as PD, PR and SD. Second outcomes were the Severe Adverse Effects (SAE) classified as grade III and IV according to Common Toxicity Criteria (CTC) v2.0 [14].

### 2.6. Statistical Analysis

Descriptive statistics were used to summarize data, with average and range for continuous variables and frequencies and percentages for nominal/dichotomous variables. The SAE were calculated as number of events per 100 and pooled in random-effects models with MetaXL (Version 5.3). One of included studies [15] in the proportion meta-analyses presented zero total event when calculating the pooled estimates as previously advised [16]. Results were considered statistically significant for a two-tailed *P* value  <  0.05.

## 3. Results

### 3.1. Study Overview

**Figure 1:**
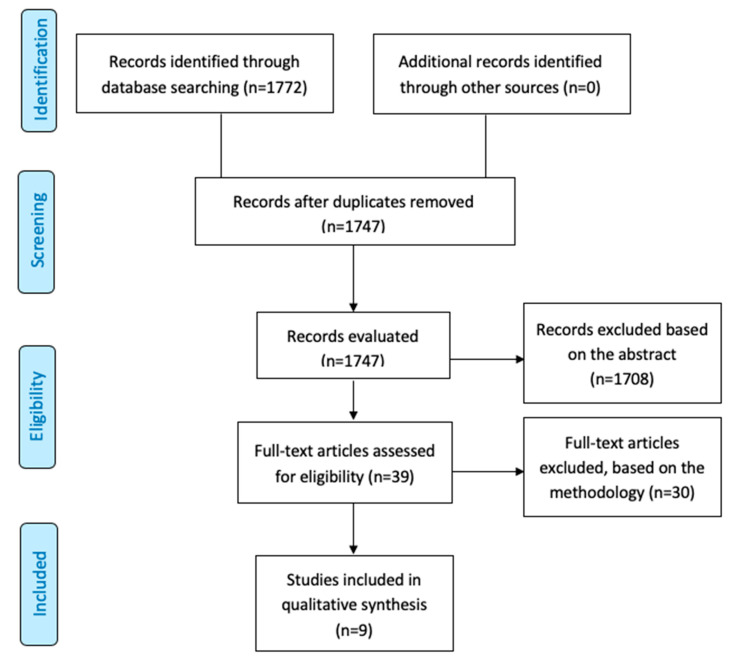
Study flow diagram. Following the guidelines of the Cochrane Handbook for Systematic Reviews of Intervention [25], it was performed a sensitive search in the online databases to identify the studies that examined associations between different nanoformulation and head and neck cancer (HNC) treatment. This systematic review searched for relevant studies considering publications up to June 2020. The chart diagram was reproduced from Moher, Preferred reporting items for systematic reviews and meta-analyses: the PRISMA statement; published by PLoS Med., 2009 [26].

A total of 1772 articles were identified. Following the exclusion of duplication or reports unrelated to cancer and/or nanoformulation, 1747 manuscripts were retrieved (Figure 1). An additional 1708 studies were excluded, as they were either abstracts or irrelevant studies regarding nanoformulation based on target therapy in HNC, leaving 39 studies for further full-text evaluation. From these, 30 studies were removed from the analysis because they did not match with inclusion criteria. The reasons for inclusion and exclusion after screening are listed in the Supplementary Tables S1 and S2. Thus, the qualitative and quantitative analysis was conducted with nine clinical studies (Phases I or II) involving nanoformulations as the administration mode of antineoplastic therapies in HNC [15,17,18,19,20,21,22,23,24].

The clinical characteristics of the nine studies included in the analysis were retrieved (Table 1). The mean age of the patients was 59.23 years (range 25–87 years) and the majority of the cases were observed in males (78.16%). Most patients had previously non-treated lesions (62.1%) and non-distant metastases (89.14%) at the moment of the diagnosis. However, the majority of the cases were advanced HNC (92.56% T3 + T4) presenting metastasis in the lymph nodes (72.48%). Of the nine manuscripts exploring nanoparticles in HNC, four of them were classified at Phase I (44.4%) and five at Phase II (55.6%) non-randomized controlled clinical trials. The anatomic location was predominantly the oral cavity (n = 97, 56.7%), followed by larynx (n = 43, 25.1%), oropharynx (n = 38, 22.2%), hypopharynx (n = 28, 16.4%), maxillary sinus (n = 16, 9.4%) and other mixed sites (n = 16, 9.4%-including nasopharynx, oropharynx, paranasal sinuses, maxillary sinus, oral cavity, and hypopharynx) (Table 1).

### 3.2. Interventions in HNC Using Chemotherapy Nanoformulations

Nanoparticles carrying different chemotherapy drugs were identified in nine studies and the information about each intervention was compiled in the Table 2. Among the nanoparticles carrier chemotherapy (NCC) categories translated into clinical trials, the liposomes were the most common complex observed, representing 6 among 9 papers, followed by 3 papers related to albumin-bound chemotherapeutics. Cisplatin (2/9), Doxorubicin (3/9) and Paclitaxel (4/9) were the chemotherapeutic agents used in these nine studies. The posology, the administration mode and the timing of intervention were very heterogeneous among the studies. The concentrations of NCC solutions were not possible to calculate, as most of the articles did not use a standardized system to report their concentrations.

### 3.3. Tumor Response and Host Toxicity

Randomized clinical trials aiming to evaluate NCC for HNC therapy were not identified in the literature. The single arms clinical trials at Phases I and II, included in the analysis, were summarized by tumor response (Table 2) and toxicity outcomes (Table 3). These studies showed small sample size (range 7–60) without control groups. It was not possible to evaluate survival outcomes with the Kaplan–Meier method and log-rank test, because these studies presented short period of follow-up (range 0.5–36 months) or the HNC patients underwent to definitive treatment after administration of NCCs.

The chemotherapies agents were heterogeneous among the articles. These nine studies used a range of different methods to assess tumor response and the adverse side effects of NCC in patients with HNC. Normally, the tumor response was evaluated in different time points in accordance with the criteria specified by World Health Organization (WHO) guidance. The treatment duration was also variable among the studies and was estimated either based on the maximum number of cycles allowed per patient, but it did not consider the progression-free survival. The most common methods used to demonstrate toxicity were the National Cancer Institute (NCI) Common Toxicity Criteria and the WHO toxicity criteria. The studies escalated NCC dose according to the chemotherapeutic drug applied until a maximum dose allowed. All these variants being evaluated thorough different criteria can introduce bias into the demonstration of the efficiency in the treatment.

**Figure 2:**
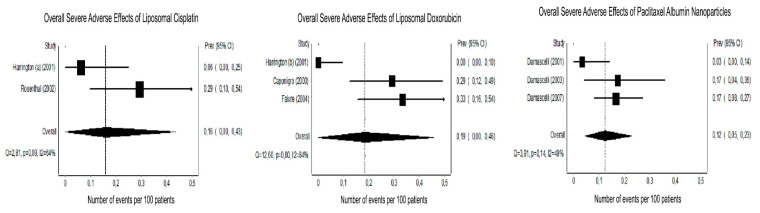
Forest plots showing the proportions of overall severe adverse events (SAE—grade 3 or 4). The graphs display the reference of the study with the name of the author and year of publication, the prevalence (Prev) and the 95% confidence interval (95% CI) for each of the three nanoparticles carrier chemotherapy (NCC) identified on the search. The overall rates of adverse events per 100 patients were calculated with random-effects models due to limited sample size.

Serious adverse effects (SAE - grade 3 or 4) were expressed as events per 100 (Table 3; Figure 2). The number of clinical trials included in this analysis ranged from 2 to 3 depending on the type of NCC investigated. One study was removed of the meta-analysis [24] because of the liposomal paclitaxel differs from albumin formulations presented by the other studies. For the meta-analysis, the forest plots showed non-significant and moderate heterogeneity between trials for the liposomal cisplatin (*P* = 0.09 and I^2^ = 64%) and paclitaxel albumin nanoparticles (*P* = 0.14 and I^2^ = 49%). The liposomal doxorubicin group displayed significant and substantial heterogeneity (*P* = 0.001 and I^2^ = 89%) (Figure 2).

### 3.4. Nanoformulation and Chemotherapeutic Agents Used in the Clinical Trials for HNC

#### 3.4.1. Platinum-Based Chemotherapy

The platinum agents cisplatin and carboplatin are used both as single agents and to form the backbone for most combination regimens in HNC. In this review, nanoparticles were combined with platinum-based chemotherapy in two studies. Harrington et al. [15] treated ten patients with cisplatin combined with liposomes (200 mg/m^2^) using two cycles every three weeks. Because of the lack of toxicity, the last eight patients received 260 mg/m^2^ every three weeks. Although the drug was well tolerated and the adverse effects were minimum, the partial response was only 11.1%. The high stability of the liposome may explain the lack of efficacy, once the slow drug release kinetics reduces the cisplatin bioavailability in the body. Thus, the drug concentration fails to exceed the threshold for therapeutic effects in patients. In another study, Rosenthal et al. [17] treated 17 patients with cisplatin combined with liposomes concurrently with radiotherapy (60–72 Gy in 6–7 weeks). The dose was escalated from 20–200 mg/m^2^ in six dose levels intravenously injected every two weeks. The estimated overall survival rate was 41% and disease-free survival was 25%. Among the adverse effects, liver toxicity or rash occurred in two patients. In addition, one patient showed elevated transaminases and neutropenia. Both studies observed low severe toxicities even at the highest cisplatin doses. It may be explained by the prolonged half-life of liposomal chemotherapy agents. Even though the first clinical trial [15] demonstrated a lack of efficacy, the second study [17] showed high therapeutic potential probably because of the association with radiotherapy.

#### 3.4.2. Doxorubicin

Doxorubicin is an anthracycline drug for which the major side effect associated with its use is the cardiotoxicity. Our study identified three clinical trials that used doxorubicin combined with NCC. Harrington et al. [22] analyzed 18 patients after intravenous infusion of doxorubicin combined with liposomes. Consecutive groups of three patients received escalating doses starting at 10 mg/m^2^ and increasing through 15 mg/m^2^ to 20 mg/m^2^. The partial response to this treatment was observed in 57% of patients without severe side effects. Caponigro et al. [18] analyzed 24 patients submitted to neoadjuvant therapy with radiation and/or chemotherapy using doxorubicin combined with pegylated liposome. The compound was administered at the initial dose of 30 mg/m^2^ and subsequently escalated by 5 mg/m^2^ per step. Partial response was 33% (95% CI: 16–55%), which is similar to the doxorubicin as a single agent. Three patients develop severe adverse effects (grade 3 and 4) with stomatitis, neutropenia, and 14 showed skin toxicity. The study conducted by Faivre et al. [19] analyzed 24 patients who received doxorubicin conjugated with pegylated liposomes by intravenous infusion at an initial dose of 35 mg/m^2^, every three weeks. In the first stage of the study, 15 patients received a dose of 35 mg/m^2^ every three weeks and 11 patients were treated with 45 mg/m^2^ of the drug. Four patients showed complete clinical response (17%; 95% CI 0.5–32%). The time observed for disease-free survival and overall survival were 3.5 and 4.6 months, respectively. Two patients showed severe adverse effects such neutropenia, however none of them had skin, digestive, cardiac or hepatic toxicities. This study shows that the high concentration of drugs increases severe adverse effects but not necessarily improve the efficacy of the clinical response.

#### 3.4.3. Paclitaxel

Paclitaxel (know as Taxol) is a microtubule-stabilizing drug that induces mitotic arrest, which leads to cell death. However, recent evidence demonstrates that intratumoral concentrations of single paclitaxel are too low to cause mitotic arrest and result in multipolar divisions instead. In our review, four clinical trials used paclitaxel associated with nanoparticles to increase drug efficacy. Damascelli et al. [23] evaluated 29 patients undergone to three treatment cycles and four-weeks interval using paclitaxel conjugated to albumin nanoparticles administered by percutaneous catheterization of the neck vessels. The starting dose of 120 mg/m^2^ was progressively increased by 30 mg/m^2^ at each subsequent level. The dose-limiting toxicity was myelosuppression. Three patients had complete clinical responses and nineteen partial responses (six previously treated patients and 13 not previously treated). In another Phase I clinical trial conducted by the same group, Damascelli et al. [21] analyzed 23 previously untreated HNC patients with paclitaxel conjugated albumin nanoparticles with the same posology. Eighteen patients (78%) had a clinical and radiologic response (complete: 26%; partial: 52%), three patients (13%) had stable disease and two cases (9%) showed disease progression. The adverse effects were hematologic (grade 3) in two patients (8.6%) and neurologic (grade 4) in two patients. These scientists in 2007 expanded the study to 60 patients in a Phase II clinical trial [20], using an initial dose of 230 mg/m^2^ and subsequently a reduced dose of 150 mg/m^2^ of paclitaxel bounded albumin nanoparticles. Complete or partial responses were observed in 45 of 60 treated patients (75%). Seven patients (11.67%) had stable disease and eight (13.33%) showed disease progression. High-grade bone marrow depression was rare, however, the reduction in the dose eliminated this specific toxicity without losing efficacy. Strieth et al. [24] performed a Phase I/II clinical trial and analysed seven HNC patients previously exposed to surgery and/or radio-chemotherapy. They were treated with paclitaxel in a liposome formulation and, after three infusions of 0.55 mg/kg or 1.1 mg/kg, the tumor volume revealed stable disease in four cases and the disease progressed in only one patient. The applied doses in liposomal formulation are far below the doses of conventional paclitaxel usually given in clinical practice, which may also be a reason for the favourable safety profile. Mild adverse events were observed, such as fatigue, chills and hypertension. These clinical trials showed evidences that paclitaxel nanoformulation has lower systemic toxicity compared with the other clinical trials testing free formulations of paclitaxel. Unfortunately, none of these studies showed a proper control group.

### 3.5. Ongoing Clinical Trials

Seven clinical trials at Phases I and II evaluating the potential of innovative nanomaterials for antineoplastic drugs release are registered in the ClinicalTrials.gov (Table 4). Most of these studies used paclitaxel as chemotherapeutic and albumin as carrier. Among these trials, none of them were designed as Phase III or includes immunotherapy and/or target-therapeutic agents.

### 3.6. Outcomes

**Figure 3:**
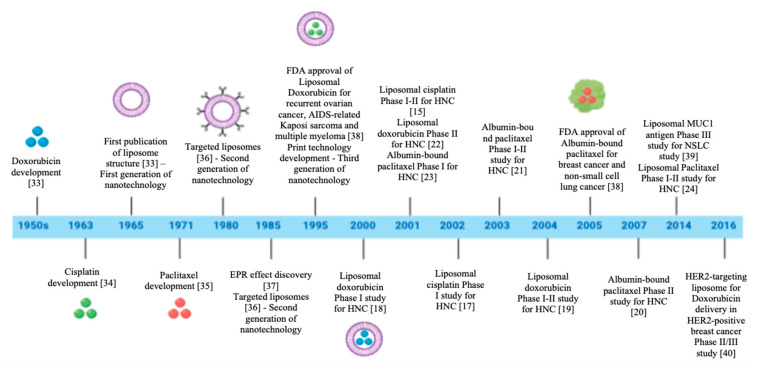
Timeline of development of nanoformulations identified in this study [15,17,18,19,20,21,22,23,24,33,34,35,36,37,38,39,40]. The scheme compiles the main chemotherapeutic agents and nanostructures for cancer treatment over the last 7 decades. The year listed in the blue line shows the moment that FDA approved the nanoformulation to the clinical practice and the intersection of the nine clinical studies analyzed in this research. NSLC: Non-Small Cell Lung Cancer; HNC: Head and Neck Cancer.

**Figure 4:**
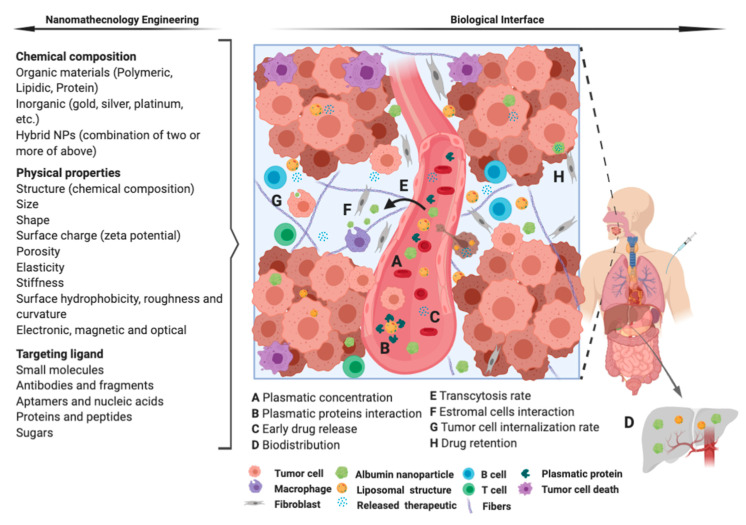
Representation of the most common types of nanoformulations identified in clinical studies of HNC. The nanoformulations carrying chemotherapeutics are expected perform transcytosis throughout the endothelium barrier and accumulate in tumor sites due the EPR effect and release the drug inside the cancer cells. This ideal pharmacokinetic depends on the nanotechnology engineering (chemical composition, physical properties and targeting ligand) and how this variables interacts with the biological events, e.g., plasmatic concentration depending on the administration mode (**A**), plasmatic proteins interaction (**B**), particle stability and drug release (**C** and **H**), biodistribution (**D**), transcytosis rate (**E**), stromal cells interaction (**F**) and tumor cell internalization (**G**).

Nanoformulation may have a significant impact in the future of oncology treatment due to the potential to improve efficacy while reduce the toxicities by enhancing drug stability, solubility and bioavailability. Such properties are motivating several therapeutic nanoproducts to move ahead towards clinical development in the last few years [27,28]. However, the majority of nanoformulations tested in HNC oncology have been at the preclinical stage. The clinical translation is not a reality yet, because there is still a need to show more evidences about the efficacy and safety. In this study, we systematically reviewed the published manuscripts about HNC nanotechnology-based therapies in clinical stages and all the current ongoing clinical trials registered on ClinicalTrials.gov. After carefully screening, it was not possible identify any randomized clinical trial testing nanoformulations of chemotherapeutics or the combination with immunotherapeutics agents for HNC treatment. These clinical trials used single arm (Phases I and II) to determine only the tumor response or adverse effects. It was noticed that some challenges, such as the achievement of the optimal combination of physic-chemical parameters to specifically target the tumor site and control drug release, are the key factors that are preventing the translation of nanomedicines into therapy [8,29]. However, global efforts have focused on the development of functional nanoparticles to increase the bioavailability in the tumor site. Several modifications were done along the last decades characterizing four generations of nanoformulation-mediated co-delivery of small-molecule chemotherapy (Figure 3). The first generation includes materials with passive drug-release mechanisms (e.g., coating, nanoparticles of polymers, metals or ceramics); the second generation comprises targeted and bioactive devices with active mechanism of drug-delivery; and the third and fourth generations consist of guided assembly and molecular nanoparticles (Figure 3). All the clinical studies included in this review tested nanoformulations for combination therapy belonging to the first generation of nanotechnology. These clinical studies investigated three conventional drugs (cisplatin, doxorubicin and paclitaxel) and excipients (albumin, lipids by itself or associated with polymers). The single use of these methods are approved by FDA and largely used in clinical practice, so there is no concern about the safety and toxicity of the excipients themselves [30]. However, these approved drugs are often obsolete technology when compared with the recent options under preclinical and clinical testing [31]. The most common types of nanoformulations, including the chemical composition, physical properties and target ligands that affect the biological processes involved in the drug delivery [32] to the tumor tissues identified in clinical studies of HNC are presented in the Figure 4.

## 4. Remarks and Future Perspectives

This systematic review and meta-analysis reveals the recent progress in the development of targeted nanoparticle systems for HNC therapy. In HNC, chemotherapy is usually used alongside surgery and/or radiotherapy in advanced cases generating severe side effects and poor quality of life. The most common chemotherapeutic agents used are platinum-based drugs (cisplatin or carboplatin) and combinations with taxanes (e.g., docetaxel) or 5-fluorouracil. However, conventional delivery methods of chemotherapeutic agents have several limitations: Firstly, some drugs have poor solubility and low bioavailability and contain toxic solvents in their formulation. Secondly, they have a short circulation time because of their physiological instability, degradation, and clearance. Thirdly, the non-specific distribution of the drugs limits the concentration achieved in the tumor and causes harmful side effects because of their unwanted accumulation in healthy tissues. A combination of chemotherapeutic agents improved drug response for patients with advanced HNC but no effect on overall survival was observed. Therefore, advanced drug delivery systems based on nanotechnology and a tumor-targeted strategy, hold considerable potential to enhance chemotherapeutic efficacy, representing a hot topic in cancer therapy for future investigations. Even though most approaches are still in the preclinical stages, they have shown tremendous potential to fulfill the need for viable alternative cancer therapies. Further researches into higher-specificity tumor targets and more efficient NCC are needed, including complex modifications to enhance the antitumor efficacy in order to achieve the ultimate goal of personalized medicine.

The studies involving nanoformulations for HNC therapy demonstrated the difficulty and limitation to demonstrate efficacy in tumor response due to the lack of clinical studies with proper gold standard controls. Besides, the short-term follow-up and the use of co-concurrent therapies, such as radiotherapy, generate bias to determine the real impact of these strategies in the success of the treatment. However, in general, all the studies showed that nanotechnologies were not associated with increased SAE in HNC. We conclude that this topic demands future and well-designed experimental studies with proper randomized clinical trials.

## Figures and Tables

**Table 1:** Clinical characteristics of the nine included clinical studies of head and neck cancer and chemotherapeutic nanoformulations.

Patient Characteristics	n
Mean age	59.23 (range 25–87) years
Total patients recruited	229 (range 7–60)
Number of patients analyzed	216 (94.3%)
Gender	
Male	179 (78.17%)
Female	50 (21.83%)
Previously treated	
Yes	88 (37.9%)
No	141 (62.1%)
Tumor Size *	
T1 + T2	11 (7.43%)
T3 + T4	137 (92.57%)
Lymph nodes metastasis *	
Positive	108 (72.48%)
Negative	41 (27.52%)
Distant metastasis *	
Yes	19 (10.86%)
No	156 (89.14%)
Died of disease *	
Yes	61 (44.20%)
No	77 (55.80%)
Tumor Location	
Oral cavity	97 (41.99%)
Larynx	43 (18.62%)
Oropharynx	38 (16.45%)
Hypopharynx	28 (12.12%)
Maxilary sinus	9 (3.89%)
Others	16 (6.92%)

* Only reported data were recorded.

**Table 2:** Clinical trials at Phases I and II investigating nanoformulations based on chemotherapy in patients with head and neck cancer.

Study (Year)	Phase	Number of Participants/ Number Analyzed	CT	Vehicle/Carrier	Dose	Previous Treatments	Median Follow-Up (Months) ^a^/ Definitive Treatment	Tumor Response ^b^ (%)		
								CR	SD	PD
Harrington (a) (2001) [15]	I-II	18/16	Cisplatin	Pegylated Liposome	2 cycles of 200 mg/m^2^ every 3 weeks. The last 8 patients received 260 mg/m^2^	No	17/RT after the second dose	11.1	55.6	33.3
Rosenthal (2002) [17]	I	20/17	Cisplatin	Pegylated Liposome	Escalated from 20–200 mg/m^2^ in six dose levels	Yes	36/Concurrent with RT	40	0	60
Harrington (b) (2001) [22]	II	20/18	Doxorubicin	Pegylated Liposome	Escalated doses starting at 10 mg/m^2^ and increasing through 15 mg/m^2^ to 20 mg/m2.	No	13/RT began after the last dose	57	31	13
Caponigro (2000) [18]	I	24/24	Doxorubicin	Pegylated Liposome	Initial dose of 30 mg/m^2^ and subsequently escalated by 5 mg/m^2^	Yes	1/Not stated	33.33	62.5	4.16
Faivre (2004) [19]	I-II	26/24	Doxorubicin	Pegylated Liposome	15 patients received a dose of 35 mg/m^2^ every 3 weeks. The following 11 patients group was treated at 45 mg/m^2^.	Yes	34/Not stated	17	33	50^d^
Damascelli (2001) [23]	I	31/28	Paclitaxel	Albumin nanoparticle	Starting dose of 120 mg/m^2^ was increased by 30 mg/m^2^ during three treatment cycles.	Yes	3-13/Not stated	75.85	17.24	6.88
Damascelli (2003) [21]	I	23/23	Paclitaxel	Albumin nanoparticle	Starting dose of 120 mg/m^2^ was increased by 30 mg/m^2^ at 3 subsequent levels each 4 weeks.	No	5-12/Not stated	78	13	9
Damascelli (2007) [20]	II	60/60	Paclitaxel	Albumin nanoparticle	Starting dose of 230 mg/m^2^ and subsequently a reduced dose of 150 mg/m^2^.	No	0.5/Surgery, CT and/or RT	75	11.67	13.33
Strieth (2014) [24]	I-II	07/05	Paclitaxel	Liposome	One group received 3 infusions of 0.55 mg/kg and another received 1.1 mg/kg.	Yes	0.75/Not stated	0	80	20

^a^ Median of follow-up was recorded after the last dose. ^b^ Tumor response on the last follow-up was recorded. ^c^ Overall survival 41% and Disease-Free Survival 25%. ^d^ Assumed value. ^e^ 1 patient developed massive necrosis and was considered. Clinical response (CR), stable disease (SD), progressive disease (PD), Radiotherapy (RT) and Chemotherapy (CT).

**Table 3:** Severe adverse events (Grade 3 or 4) in patients receiving nanoformulations based on chemotherapy for head and neck cancer.

Study	CT and Vehicle	Number of Analyzed Patients	Main Severe Adverse Effects (n)					
			Hematological	Neutro-/Leucopenia	Gastrointestinal ^b^	Mucocutaneous	Neurological	Allergy
Harrington [15]	Cisplatin (Liposome)	16	1					
Rosenthal [17]	Cisplatin (Liposome)	17	2	1	2			
Total (events per 100 patients)	..	33	3 (9)	1 (3)	2 (6)	0	0	0
Harrington [22]	Doxorubicin (Liposome)	18						
Caponigro [18]	Doxorubicin (Liposome)	24		2		5		
Faivre [19]	Doxorubicin (Liposome)	24	3	2	1			2
Total (events per 100 patients)	..	66	3 (5)	4 (6)	1 (2)	5 (8)	0	2 (4)
Damascelli [23]	Paclitaxel (Albumin nanoparticle)	29		1				
Damascelli [21]	Paclitaxel (Albumin nanoparticle)	23	2				2	
Damascelli [20]	Paclitaxel (Albumin nanoparticle)	60		4			6	
Strieth [24]	Paclitaxel (Liposome)	05						
Total (events per 100 patients)	..	117	2 (2)	5 (4)	0	0	8 (7)	0

^a^ Only Severe Adverse Effects (SAE) classified as grade III and IV according to Common Toxicity Criteria (CTC) v2.0 were recorded. ^b^ Gastrointestinal events include diarrhoea, constipation, vomiting, nausea, anorexia, gastrointestinal perforation and gastrointestinal bleeding, bilirubin elevation, alkaline phosphatase elevation. Cells are left empty when a study did not report on an adverse event.

**Table 4:** List of ongoing clinical trials in head and neck cancer using nanoformulations (source: ClinicalTrials.gov).

Phase	Year	NCC	Identifier Number
I	2016	Cisplatin polymeric micelle	NCT02817113
I	2013	Paclitaxel albumin- nanoparticle	NCT01847326
I	2008	Paclitaxel albumin- nanoparticle	NCT00736619
II	2014	Paclitaxel albumin- nanoparticle	NCT02033538
II	2009	Paclitaxel albumin- nanoparticle	NCT00851877
II	2012	Paclitaxel albumin- nanoparticle	NCT01566435
na *	2007	Paclitaxel albumin- nanoparticle	NCT00499291

* na: not applicable.

## Supplementary Materials

The following are available online at https://www.mdpi.com/2079-4991/10/10/1938/s1, Supplementary Table S1: Characteristics of included studies after full text screening, Supplementary Table S2: Characteristics of the excluded studies after full text screening. Supplementary Material S1—Endnote Library.
