# Supplementary material for: Nanoparticle-Based Chemotherapy Formulations for Head and Neck Cancer: A Systematic Review and Perspectives
Source: Nanomaterials (Basel). 2020 Sep 29;10(10):1938. doi: 10.3390/nano10101938 (PMC7600408; doi:10.3390/nano10101938)
Supplement: Supplementary file 1 [file nanomaterials-10-01938-s001.pdf]

SUPPLEMENTAL MATERIAL

Supplementary Table S1. Characteristics of included studies after full text screening.

Nanoparticle-Based Chemotherapy Formulations for Head and Neck Cancer: A Systematic Review and Perspectives

Jefferson Muniz de Lima [1,2,3], Paulo Rogerio Bonan [3], Danyel Elias da Cruz Perez [4], Michael Hier [1], Moulay A. Alaoui-Jamali [2] and Sabrina Daniela da Silva [1,2,*]

1 Department of Otolaryngology Head and Neck Surgery, Sir Mortimer B. Davis-Jewish General Hospital, McGill University, Montreal, QC, H3T 1E2, Canada; jefferson.idalino@gmail.com (J.M.L.); mhier@jgh.mcgill.ca (M.H.)
2 Segal Cancer Centre and Lady Davis Institute for Medical Research, Sir Mortimer B. Davis-Jewish General Hospital, Departments of Medicine, Oncology, and Pharmacology and Therapeutics, Faculty of Medicine, McGill University, Montreal, QC, H3T 1E2, Canada; moulay.alaoui-jamali@mcgill.ca;
3 Federal University of Paraiba (UFPB), Department of Clinical and Social Dentistry, Faculty of Dentistry, Joao Pessoa, PB, 58051-900, Brazil; pbonan@yahoo.com
4 Universidade Federal de Pernambuco, Department of Clinical and Preventive Dentistry, School of Dentistry, Recife, PE, 50740-521, Brazil; danyel.perez@ufpe.br
* Correspondence: sabrina.wurzba@gmail.com

Appendix A - Search Strategy

MEDLINE via OVID search strategy

1. "Mouth Neoplasms"/ or "Head and Neck Neoplasms"/ or "Gingival Neoplasms"/ or "Palatal Neoplasms"/ or "Tongue Neoplasms"/
2. ((cancer$ or tumour$ or tumor$ or neoplas$ or malignan$ or carcinoma$ or metatasta$) adj5 (oral$ or intra-oral$ or intraoral$ or "intra-oral$" or gingiva$ or oropharyn$ or mouth$ or tongue$ or cheek$ or gum$ or palatal$ or palate$ or "head and neck")).ti,ab.
3. ((cancer$ or tumour$ or tumor$ or neoplas$ or malignan$ or carcinoma$ or metatasta$) adj5 (oral$ or intra-oral$ or intraoral$ or "intra-oral$" or gingiva$ or oropharyn$ or mouth$ or tongue$ or cheek$ or gum$ or palatal$ or palate$ or "head and neck")).ti,ab.
4. 1 or 2 or 3
5. exp antineoplastic agents/ or drug therapy/ or exp antineoplastic protocols/
6. ((angiogenes$ or anticarcinogenic$ or antimetabolite$ or antimitotic$ or antineoplastic$ or alkylat$ or hormonal$ or phytogenic$ or immunologic$ or myeloablative$ or adp-ribose polymerase$ or topoisomerase$) adj3 (inhibitor$ or agent$ or antineoplastic$)).ti,ab.
7. 5 or 6
8. drug delivery systems/ or exp drug carriers/ or exp drug liberation/ or exp dendrimers/ or exp nanocapsules/ or exp nanoconjugates/ or exp nanostructures/ or exp nanocomposites/ or exp nanofibers/ or exp nanoparticles/ or exp dendrimers/ or exp metal nanoparticles/ or exp magnetite nanoparticles/ or exp nanoshells/ or exp nanocapsules/ or exp nanoconjugates/exp or exp nanodiamonds/ or exp nanospheres/ or exp quantum dots/ or exp nanopores/ or exp nanotubes/ or exp nanowires/
9. (nanotechnolog$ or "nanomedicine nanostructure$" or nanocomposite$ or nanofiber$ or nanoparticle$ or dendrimer$ or metal nanoparticle$ or magnetite nanoparticle$ or nanoshell$ or nanocapsule$ or nanoconjugate$ or nanodiamond$ or nanosphere$ or quantum dot$ or

nanopore$ or nanotube$ or nanowire$ or nanomaterial& or nabiomaterial$ or nanoformula$).ti,ab.
10. drug$ adj3 (delivery system$ or release$ release control$ carrier$).ti,ab.
11. 8 or 9 or 10

The query above was combined with the Medline OVID filter from Cochrane - Max Sensitivity for identifying randomized trials as referred on Cochrane Handbook for Systematic Reviews of Interventions version 5.1.0 [30].

1. randomized controlled trial.pt.
2. controlled clinical trial.pt.
3. randomized.ab.
4. placebo.ab.
5. drug therapy.fs.
6. randomly.ab.
7. trial.ab.
8. groups.ab.
9. or/1-8
10. exp animals/ not humans.sh.
11. 9 not 10

EMBASE via OVID search strategy

1. mouth cancer/ or mouth carcinoma/ or "head and neck carcinoma"/ or mouth squamous cell carcinoma/ or tongue carcinoma/ or tonsil carcinoma/
2. ((cancer$ or tumour$ or tumor$ or neoplas$ or malignan$ or carcinoma$ or metatasta$) adj5 ((oral$ or intra-oral$ or gingiva$ or oropharyn$ or mouth$ or tongue$ or cheek$ or gum$ or palat$ or intraoral or head) and neck)).ti,ab.
3. 1 or 2
4. exp antineoplastic activity/ or exp antineoplastic agent/
5. (chemotherap$ or anticarcinogen$ or anticancerogen$ or antineoplastic$ or anti neoplastic$ anticancer$ or carcinostatic$).ti,ab.
6. (anticancer or anti cancer).ti,ab.
7. ((tumor or tumour) adj2 inhibitor).ti,ab.
8. (anti tumor or antitumor or antitumou or anti tumour).ti,ab.
9. 4 or 5 or 6 or 7 or 8
10. exp drug delivery system/ or exp drug carrier/ or exp drug delivery device/ or exp liposomal delivery/ or exp sustained release preparation/ or exp nanobead/ or exp nanobiotechnology/ or exp nanocapsule/ or exp nanocarrier/ or exp nanocatalysis/ or exp nanocoating/ or exp nanoconjugate/ or exp nanocrystal/ or exp nanodevice/ or exp nanodiamond/ or exp nanodisc/ or exp nanoemulsion/ or exp nanoencapsulation/ or exp nanoengineering/ or exp nanofabrication/ or exp nanofiber/ or exp nanofilm/ or exp nanomaterial/ or exp nanomedicine/ or exp nanoparticle/ or nanopharmaceutics/ or exp nanopore/ or exp nanorope/ or exp nanosheet/ or exp nanoshell/ or exp nanosphere/ or exp nanotechnology/
11. (nanotechnolog$ or "nanomedicine nanostructure$" or nanocomposite$ or nanofiber$ or nanoparticle$ or dendrimer$ or metal nanoparticle$ or magnetite or nanoparticle$ or nanoshell$ or nanocapsule$ or nanoconjugate$ or nanodiamond$ or nanosphere$ or quantum dot$ or nanopore$ or nanotube$ or nanowire$ or nanomaterial$ or nanobiomaterial$ or nanoformula$).ti,ab.
12. 10 or 11

The query above was combined with the Cochrane Oral Health Group's RCT filter for searching EMBASE via Ovid (www.cochranelibrary.com/help/central-creation-details.html for information):

1. Randomized controlled trial/

2. Controlled clinical study/
3. Random$.ti,ab.
4. randomization/
5. intermethod comparison/
6. placebo.ti,ab.
7. (compare or compared or comparison).ti.
8. ((evaluated or evaluate or evaluating or assessed or assess) and (compare or compared or comparing or comparison)).ab.
9. (open adj label).ti,ab.
10. ((double or single or doubly or singly) adj (blind or blinded or blindly)).ti,ab.
11. double blind procedure/
12. parallel group$1.ti,ab.
13. (crossover or cross over).ti,ab.
14. ((assign$ or match or matched or allocation) adj5 (alternate or group$1 or intervention$1 or patient$1 or subject$1 or participant$1)).ti,ab.
15. (assigned or allocated).ti,ab.
16. (controlled adj7 (study or design or trial)).ti,ab.
17. (volunteer or volunteers).ti,ab.
18. human experiment/
19. trial.ti.
20. or/2-19
21. 20 not 1
22. (random$ adj sampl$ adj7 ("cross section$" or questionnaire$1 or survey$ or database$1)).ti,ab. not (comparative study/ or controlled study/ or randomi?ed controlled.ti,ab. or randomly assigned.ti,ab.)
23. Cross-sectional study/ not (randomized controlled trial/ or controlled clinical study/ or controlled study/ or randomi?ed controlled.ti,ab. or control group$1.ti,ab.)
24. (((case adj control$) and random$) not randomi?ed controlled).ti,ab.
25. (Systematic review not (trial or study)).ti.
26. (nonrandom$ not random$).ti,ab.
27. "Random field$".ti,ab.
28. (random cluster adj3 sampl$).ti,ab.
29. (review.ab. and review.pt.) not trial.ti.
30. "we searched".ab. and (review.ti. or review.pt.)
31. "update review".ab.
32. (databases adj4 searched).ab.
33. (rat or rats or mouse or mice or swine or porcine or murine or sheep or lambs or pigs or piglets or rabbit or rabbits or cat or cats or dog or dogs or cattle or bovine or monkey or monkeys or trout or marmoset$1).ti. and animal experiment/
34. Animal experiment/ not (human experiment/ or human/)
35. or/22-34
36. 21 not 35

The Cochrane Central Register of Controlled Clinical Trials (CENTRAL) search strategy

#1 MeSH descriptor: [undefined] explode all trees
#2 MeSH descriptor: [Squamous Cell Carcinoma of Head and Neck] this term only
#3 MeSH descriptor: [Mouth Neoplasms] this term only
#4 MeSH descriptor: [D005887] explode all trees
#5 MeSH descriptor: [D010157] explode all trees
#6 MeSH descriptor: [D014062] explode all trees
#7 (((oral in Title, Abstract or Keywords near/6 cancer* in Title, Abstract or Keywords) or (oral in Title, Abstract or Keywords near/6 tumour* in Title, Abstract or Keywords) or (oral in

Title, Abstract or Keywords near/6 tumor* in Title, Abstract or Keywords) or (oral in Title, Abstract or Keywords near/6 neoplas* in Title, Abstract or Keywords) or (oral in Title, Abstract or Keywords near/6 malignan* in Title, Abstract or Keywords) or (oral in Title, Abstract or Keywords near/6 carcinoma* in Title, Abstract or Keywords) or (oral in Title, Abstract or Keywords near/6 metastas* in Title, Abstract or Keywords) or (cancer* in Title, Abstract or Keywords near/6 intra-oral* in Title, Abstract or Keywords) or (intra-oral* in Title, Abstract or Keywords near/6 tumour* in Title, Abstract or Keywords) or (intra-oral* in Title, Abstract or Keywords near/6 tumor* in Title, Abstract or Keywords) or (intra-oral* in Title, Abstract or Keywords near/6 neoplas* in Title, Abstract or Keywords) or (intra-oral* in Title, Abstract or Keywords near/6 malignan* in Title, Abstract or Keywords) or (intra-oral* in Title, Abstract or Keywords near/6 carcinoma* in Title, Abstract or Keywords) or (intra-oral* in Title, Abstract or Keywords near/6 metastas* in Title, Abstract or Keywords) or (cancer* in Title, Abstract or Keywords near/6 intraoral* in Title, Abstract or Keywords) or (intraoral* in Title, Abstract or Keywords near/6 tumour* in Title, Abstract or Keywords) or (intraoral* in Title, Abstract or Keywords near/6 tumor* in Title, Abstract or Keywords) or (intraoral* in Title, Abstract or Keywords near/6 neoplas* in Title, Abstract or Keywords) or (intraoral* in Title, Abstract or Keywords near/6 malignan* in Title, Abstract or Keywords) or (intraoral* in Title, Abstract or Keywords near/6 carconoma* in Title, Abstract or Keywords) or (intraoral* in Title, Abstract or Keywords near/6 metasta* in Title, Abstract or Keywords) or (gingiva* in Title, Abstract or Keywords near/6 cancer* in Title, Abstract or Keywords) or (gingiva* in Title, Abstract or Keywords near/6 tumour* in Title, Abstract or Keywords) or (gingiva* in Title, Abstract or Keywords near/6 tumor* in Title, Abstract or Keywords) or (gingiva* in Title, Abstract or Keywords near/6 neoplas* in Title, Abstract or Keywords) or (gingiva* in Title, Abstract or Keywords near/6 malignan* in Title, Abstract or Keywords) or (gingiva* in Title, Abstract or Keywords near/6 carcinoma* in Title, Abstract or Keywords) or (gingiva* in Title, Abstract or Keywords near/6 metastas* in Title, Abstract or Keywords) or (oropharyn* in Title, Abstract or Keywords near/6 cancer* in Title, Abstract or Keywords) or (oropharyn* in Title, Abstract or Keywords near/6 tumour* in Title, Abstract or Keywords) or (oropharyn* in Title, Abstract or Keywords near/6 tumor* in Title, Abstract or Keywords) or (oropharyn* in Title, Abstract or Keywords near/6 neoplas* in Title, Abstract or Keywords) or (oropharyn* in Title, Abstract or Keywords near/6malignan* in Title, Abstract or Keywords) or (oropharyn* in Title, Abstract or Keywords near/6 carcinoma* in Title, Abstract or Keywords) or (oropharyn* in Title, Abstract or Keywords near/6 metastas* in Title, Abstract or Keywords) or (mouth* in Title, Abstract or Keywords near/6 cancer* in Title, Abstract or Keywords) or (mouth* in Title, Abstract or Keywords near/6 tumour* in Title, Abstract or Keywords) or (mouth* in Title, Abstract or Keywords near/6 tumor* in Title, Abstract or Keywords) or (mouth* in Title, Abstract or Keywords near/6 neoplas* in Title, Abstract or Keywords) or (mouth* in Title, Abstract or Keywords near/6malignan* in Title, Abstract or Keywords) or (mouth* in Title, Abstract or Keywords near/6 carcinoma* in Title, Abstract or Keywords) or (mouth* in Title, Abstract or Keywords near/6 metastas* in Title, Abstract or Keywords) or (tongue* in Title, Abstract or Keywords near/6 cancer* in Title, Abstract or Keywords) or (tongue* in Title,Abstract or Keywords near/6 tumour* in Title, Abstract or Keywords) or (tongue* in Title, Abstract or Keywords near/6 tumor* in Title, Abstract or Keywords) or (tongue* in Title, Abstract or Keywords near/6 neoplas* in Title, Abstract or Keywords) or (tongue* in Title, Abstract or Keywords near/6 malignan* in Title, Abstract or Keywords) or (tongue* in Title, Abstract or Keywords near/6 carcinoma* in Title, Abstract or Keywords) or (tongue* in Title, Abstract or Keywords near/6 metastas* in Title, Abstract or Keywords) or (cheek in Title, Abstract or Keywords near/6 cancer* in Title, Abstract or Keywords) or (cheek in Title, Abstract or Keywords near/ 6 tumour* in Title, Abstract or Keywords) or (cheek in Title, Abstract or Keywords near/6 tumor* in Title, Abstract or Keywords) or (cheek in Title, Abstract or Keywords near/6 neoplas* in Title, Abstract or Keywords) or (cheek in Title, Abstract or

Keywords near/6 malignan* in Title, Abstract or Keywords) or (cheek in Title, Abstract or Keywords near/6 carcinoma* in Title, Abstract or Keywords) or (cheek in Title, Abstract or Keywords near/6 metastas* in Title, Abstract or Keywords) or (cheeks in Title, Abstract or Keywords near/6 cancer* in Title, Abstract or Keywords) or (cheeks in Title, Abstract or Keywords near/6 tumour* in Title, Abstract or Keywords) or (cheeks in Title, Abstract or Keywords near/6 tumor* in Title, Abstract or Keywords) or (cheeks in Title, Abstract or Keywords near/ 6 neoplas* in Title, Abstract or Keywords) or (cheeks in Title, Abstract or Keywords near/6 malignan* in Title, Abstract or Keywords) or (cheeks in Title, Abstract or Keywords near/6 carcinoma* in Title, Abstract or Keywords) or (cheeks in Title, Abstract or Keywords near/6 metastas* in Title, Abstract or Keywords) or (gum in Title, Abstract or Keywords near/6 cancer* in Title, Abstract or Keywords) or (gum in Title, Abstract or Keywords near/6 tumour* in Title, Abstract or Keywords) or (gum in Title, Abstract or Keywords near/ 6 tumor* in Title, Abstract or Keywords) or (gum in Title, Abstract or Keywords near/6 neoplas* in Title, Abstract or Keywords) or (gum in Title, Abstract or Keywords near/6 malignan* in Title, Abstract or Keywords) or (gum in Title, Abstract or Keywords near/6 carcinoma* in Title, Abstract or Keywords) or (gum in Title, Abstract or Keywords near/6 metastas* in Title, Abstract or Keywords) or (gums in Title, Abstract or Keywords near/6 cancer* in Title, Abstract or Keywords) or (gums in Title, Abstract or Keywords near/6 tumour* in Title, Abstract or Keywords) or (gums in Title, Abstract or Keywords near/6 tumor* in Title, Abstract or Keywords) or (gums in Title, Abstract or Keywords near/6 neoplas* in Title, Abstract or Keywords) or (gums in Title, Abstract or Keywords near/6 malignan* in Title, Abstract or Keywords) or (gums in Title, Abstract or Keywords near/6 carcinoma* in Title, Abstract or Keywords) or (gums in Title, Abstract or Keywords near/6 metastas* in Title, Abstract or Keywords) or (palate in Title, Abstract or Keywords near/6 cancer* in Title, Abstract or Keywords) or (palate in Title, Abstract or Keywords near/6 tumour* in Title, Abstract or Keywords) or (palate in Title, Abstract or Keywords near/6 tumor* in Title, Abstract or Keywords) or (palate in Title, Abstract or Keywords near/6 neoplas* in Title, Abstract or Keywords) or (palate in Title, Abstract or Keywords near/6 malignan* in Title, Abstract or Keywords) or (palate in Title, Abstract or Keywords near/6 carcinoma* in Title, Abstract or Keywords) or (palate in Title, Abstract or Keywords near/6 metastas* in Title, Abstract or Keywords) or (palatal in Title, Abstract or Keywords near/6 cancer* in Title, Abstract or Keywords) or (palatal in Title, Abstract or Keywords near/6 tumour* in Title, Abstract or Keywords) or (palatal in Title, Abstract or Keywords near/ 6 tumor* in Title, Abstract or Keywords) or (palatal in Title, Abstract or Keywords near/6 neoplas* in Title, Abstract or Keywords) or (palatal in Title, Abstract or Keywords near/6 malignan* in Title, Abstract or Keywords) or (palatal in Title, Abstract or Keywords near/6 carcinoma* in Title, Abstract or Keywords) or (palatal in Title, Abstract or Keywords near/6 metastas* in Title, Abstract or Keywords) or ("head and neck" in Title, Abstract or Keywords near/6 cancer* in Title, Abstract or Keywords) or ("head and neck" in Title, Abstract or Keywords near/6 tumour* in Title, Abstract or Keywords) or ("head and neck" in Title, Abstract or Keywords near/ 6 tumor* in Title, Abstract or Keywords) or ("head and neck" in Title, Abstract or Keywords near/6 neoplas* in Title, Abstract or Keywords) or ("head and neck" in Title, Abstract or Keywords near/6 malignan* in Title, Abstract or Keywords) or ("head and neck" in Title, Abstract or Keywords near/6 carcinoma* in Title, Abstract or Keywords) or ("head and neck" in Title, Abstract or Keywords near/6 metastas* in Title, Abstract or Keywords))):ti,ab,kw

#8 (#1 or #2 or #3 or #4 or #5 or #6 or #7)

#9 MeSH descriptor: [D000970] explode all trees

#10 (antineoplas* or immunotherapy* or target therap* or chemotherap*):ti,ab,kw

#11 (((angiogenes* in Title, Abstract or Keywords near/6 inhibitor* in Title, Abstract or Keywords) or (anticarcinogenic* in Title, Abstract or Keywords near/6 agent* in Title, Abstract or Keywords) or (anticarcinogenic* in Title, Abstract or Keywords near/6 agent* in Title,

Abstract or Keywords) or (antimetabolite* in Title, Abstract or Keywords near/6 agent* in Title, Abstract or Keywords) or (antimitotic* in Title, Abstract or Keywords near/6 agent* in Title, Abstract or Keywords) or (antineoplastic* in Title, Abstract or Keywords near/6 agent* in Title, Abstract or Keywords) (alkylat* in Title, Abstract or Keywords near/6 antineoplastic* in Title, Abstract or Keywords) or (hormonal* in Title, Abstract or Keywords near/6 antineoplastic* in Title, Abstract or Keywords) or (phytogenic* in Title, Abstract or Keywords near/6 antineoplastic* in Title, Abstract or Keywords) or (immunologic* in Title, Abstract or Keywords near/6 antineoplastic* in Title, Abstract or Keywords))):ti,ab,kw

#12 (#9 or #10 or #11)

#13 MeSH descriptor: [Drug Carriers] explode all trees

#14 ((drug* in Title, Abstract or Keywords near/6 delivery system* in Title, Abstract or Keywords) or (drug* in Title, Abstract or Keywords near/6 release* in Title, Abstract or Keywords) or (drug* in Title, Abstract or Keywords near/6 delivery system* in Title, Abstract or Keywords) or (drug* in Title, Abstract or Keywords near/6 release control* in Title, Abstract or Keywords) or (drug* in Title, Abstract or Keywords near/6 delivery system* in Title, Abstract or Keywords carrier*)):ti,ab,kw

#15 (nanotechnolog* or nanomedicine* or nanostructure* or nanocomposite* or nanofiber* or nanoparticle* or dendrimer* or metal nanoparticle* or magnetite nanoparticle* or nanoshell* or nanocapsule* or nanoconjugate* or nanodiamond* or nanosphere* or quantum dot* or nanopore* or nanotube* or nanowire* or nanomaterial* or biomaterial* or nanoformula*):ti,ab,kw

#16 (#13 or #14 or #15)

#17 (#8 and #12 and #16)

WHO International Clinical Trials Registry Platform search strategy

mouth neoplasms or mouth cancer or head and neck neoplasms or gingival neoplasms or palatal neoplasms or tongue neoplasms and antineoplastic agents or drug therapy or antineoplastic protocols or chemotherap* or target therap* or immunotherap* and drug delivery systems or drug carriers or drug liberation or dendrimers or nanocapsules or nanoconjugates or nanostructures or nanocomposites or nanofibers or nanoparticles or dendrimers or metal nanoparticles or magnetite nanoparticles or nanoshells or nanocapsules or nanoconjugates or nanodiamonds or nanospheres or quantum dots or nanopores or nanotubes or nanowires

Web of Science search strategy

#8 #7 AND #6 AND #5 AND #2 AND #1

DocType=All document types; Language=All languages;

#7 (ts=(human* or volunteer* or pacient*)) AND IDIOMA: (English) AND TIPOS DE DOCUMENTO: (Article)

DocType=All document types; Language=All languages;

#6 (TS= clinical trial* OR TS=research design OR TS=comparative stud* OR TS=evaluation stud* OR TS=controlled trial* OR TS=follow-up stud* OR TS=prospective stud* OR TS=random* OR TS=placebo* OR TS=(single blind*) OR TS=(double blind*)) AND IDIOMA: (English) AND TIPOS DE DOCUMENTO: (Article)

DocType=All document types; Language=All languages;

#5 #4 OR #3

DocType=All document types; Language=All languages;

#4 (ts=(dendrimer* or nanocapsule* or nanoconjugate* or nanostructure* or nanocomposite* or nanofiber* or nanoparticle* or dendrimer* or metal nanoparticle* or magnetite nanoparticle* or nanoshell* or nanocapsule* or nanoconjugate* or nanodiamond* or nanosphere* or quantum dot* or nanopore* or nanotube* or nanowire*)) AND IDIOMA: (English) AND TIPOS DE DOCUMENTO: (Article)

DocType=All document types; Language=All languages;

#3 (ts=(drug delivery or drug* near system* or carrier* or liberation*)) AND IDIOMA: (English) AND TIPOS DE DOCUMENTO: (Article)
DocType=All document types; Language=All languages;
#2 (ts=(antineoplastic or drug therap* near chemotherap* or target therap* or immunotherap*)) AND IDIOMA: (English) AND TIPOS DE DOCUMENTO: (Article)
DocType=All document types; Language=All languages;
#1 (ts=(mouth or oral or head and neck or gum or palatal or tongue or cheeks near neoplas* or cancer*)) AND IDIOMA: (English) AND TIPOS DE DOCUMENTO: (Article)
DocType=All document types; Language=All languages;

CINAHL (EBSCO Version) search strategy

(mouth or oral or head and neck or gum or palatal or tongue or cheeks adj toumo* tumo* neoplas* or cancer*) AND (antineoplastic or drug therap* adj chemotherap* or target therap* or immunotherap) AND (drug delivery or drug* adj system* or carrier* or liberation*) OR (dendrimer* or nanocapsule* or nanoconjugate* or nanostructure* or nanocomposite* or nanofiber* or nanoparticle* or dendrimer* or metal nanoparticle* or magnetite nanoparticle* or nanoshell* or nanocapsule* or nanoconjugate* or nanodiamond* or nanosphere* or quantum dot* or nanopore* or nanotube* or nanowire*)

The descriptos above were combined with randomized controlled trial filter derived from the editorial team of Specialized Register, CINAHL In Cochrane Stroke Group. About The Cochrane Collaboration (Cochrane Review Groups (CRGs)) 2013, Issue 10. Art. No.: STROKE.

((MH "Random Assignment") or (MH "Random Sample+") or (MH "Crossover Design") or (MH "Clinical Trials+") or (MH "Comparative Studies") or (MH "Control (Research)+") or (MH "Control Group") or (MH "Factorial Design") or (MH "Quasi-Experimental Studies+") or (MH "Placebos") or (MH "Meta Analysis") or (MH "Sample Size") or (MH "Research, Nursing") or (MH "Research Question") or (MH "Research Methodology+") or (MH "Evaluation Research+") or (MH "Concurrent Prospective Studies") or (MH "Prospective Studies") or (MH "Nursing Practice, Research-Based") or (MH "Solomon Four-Group Design") or (MH "One-Shot Case Study") or (MH "Pretest-Posttest Design+") or (MH "Static Group Comparison") or (MH "Study Design") or (MH "Clinical Research+")) or (clinical nursing research or random* or cross?over or placebo* or control* or factorial or sham* or meta?analy* or systematic review* or blind* or mask* or trial*)

nanotechnology/ or nanomedicine/

drug delivery systems/ or drug carriers/ or dendrimers/ or nanocapsules/ or nanoconjugates/

nanostructures/ or nanocomposites/ or nanofibers/ or nanoparticles/ or dendrimers/ or metal nanoparticles/ or magnetite nanoparticles/ or nanoshells/ or nanocapsules/ or nanoconjugates/ or nanodiamonds/ or nanospheres/ or quantum dots/ or nanopores/ or nanotubes/ or nanowires/

(nanotechnolog$ or "nanomedicine" nanostructure$ or nanocomposite$ or nanofiber$ or nanoparticle$ or dendrimer$ or metal nanoparticle$ or magnetite nanoparticle$ or nanoshell$ or nanocapsule$ or nanoconjugate$ or nanodiamond$ or nanosphere$ or quantum dot$ or nanopore$ or nanotube$ or nanowire$ or nanomaterial& or nabiomaterial$ or nanoformula$).ti,ab.

((drug$) adj3 (delivery system$ or release$ release control$ carrier$)).ti,ab.

Study (year)	Methods and Level of Evidence	Participants	Interventions	Outcomes	Other Treatments	Results	Remarks
HARRINGTON (2001) a [1]	Phases I-II study<br><br>Level of evidence: IV	Inclusion criteria: biopsy-proven HNC, age 18-75 years, KPS > 60%,, treatment naive, bidimensionally assessable disease, adequate bone marrow, hepatic and renal function and informed consent.<br>Exclusion criteria: The exclusion criteria were life expectancy < 3 months, acute infection requiring systemic therapy, another primary tumour.<br>Recruitment period: not stated<br>Number of participants: 18<br>Number analyzed: 16<br>Tumor site: oropharynx (8), larynx (3), hypopharynx (5), oral cavity (1) and cervical esophagus (1)	Vehicle: hydrogenated soybean phosphatidylcholine 51%. cholesterol 44%. and N-(carbamoyl-methoxypolyethyleneglycol 2000)-l.2-dis-tearoyl-i/7-glycero-3-phospho-ethanolamine sodium salt (MPEG-DSPE) 5%<br><br>Drug: cisplatin<br><br>The first 10 patients received 2 cycles of 200 mg/m^2 every 3 weeks. Because of the lack of toxicity, the last 8 patients received 260 mg/m^2 every 3 weeks.	Tumor response: Response was evaluated by clinical and repeat radiological examination according to World Health Organization (WHO) criteria for tumor response.<br><br>Adverse effects: Toxicity assessment by hematological. biochemical and Serial glomerular filtration rate (GFR) parameters, measurement Stomatitis/mucositis and myelosuppression by National Cancer Institute (NCI) Common Toxicity Criteria.<br><br>Duration of follow-up: 17 months	Radiotherapy began 21-26 days after the second dose.	Tumor response: Clinical response 0%, Partial response 11.1% Non-response, 55.6% and Progressive disease 33.3 %.<br><br>Clinical response to RT: Clinical response 37,5%, partial response 37.5%, non-response 18.8%, and Progressive disease 6.3%.<br><br>Adverse effects: the drug was tolerated well with no haematological, renal, hepatic or neurological toxicities Nausea and vomiting were minimal Leukopenia and thrombocytopenia did not occur. No renal dysfunction. There was	The high stability of the liposome may lead to the lack of efficacy, reducing bioavailability and slow drug release kinetics. Thus the drug concentration fails to exceed the threshold for therapeutic effects in patients.

						no mucocutaneous toxicity. There were no drug-related delays in the delivery of RT RT-induced mucosal and cutaneous toxicity were not significantly increased.	
HARRINGTON (2001) b [7]	Phase II study<br><br>Level of evidence: IV	Inclusion criteria: biopsy-proven, locally advanced, inoperable SCCHN; no prior therapy; at least one lesion measureable bidimensionally by physical or radiological examination; Karnofsky Performance Score (KPS) 560%; written informed consent.<br>Exclusion criteria: life expectancy <3 months,	Vehicle: pegylated liposomal<br><br>Drug: doxorubicin<br><br>The drug was administered as a slow intravenous infusion.<br>Consecutive groups of 3 patients received escalating doses starting at 10 mg/m^2 and increasing through 15 mg/m^2 to 20 mg/m^2.	Tumor response: clinical and repeated radiological examination using the WHO criteria for tumor response. Changes in tumor volumes were calculated by reconstructing the tumor volume from computed tomography scans.<br><br>Adverse effects: Haematological and non-	Radiotherapy began after the last dose.	Tumor response: Clinical response 19%, Partial response 38%, Non-response 31% and Progressive disease 13 %.<br><br>Clinical response to RT: Clinical response 80%, Partial response 13%, Non-response 0% and Progressive disease 7%.	The compound had significant activity against SCCHN and warrants further investigation in this disease. In view of its tumour targeting properties and activity at moderate doses, it may be useful in concomitant chemoradiotherapy strategies for SCCHN.

		acute infection requiring systemic therapy, another primary tumour.<br>Recruitment period: not stated<br>Number of participants: 20<br>Number analysed: 18<br>Tumor site: oropharynx (8), larynx (5), hypopharynx (6) and oral cavity (1)		haematological toxicities were assessed during the initial two cycles.<br>Cardiac function was accessed by electrocardiogram (ECG) and nuclear medicine MUGA scan. Stomatitis/mucositis, palmarplantar erythrodysaesthesia and myelosuppression were recorded using the NCI Common Toxicity Criteria<br><br>Duration of follow-up: 34 months		Adverse effects:<br>There was no grade 3/4 hematological, mucosal or cardiac toxicity.<br>Nausea and vomiting were minimal. There were no drug-related RT delays. Local RT-induced toxicity was not increased.	
Damascelli (2003)<br>[6]	Phase I study<br><br>Level of evidence: IV	Inclusion criteria: Previously untreated patients with a histologic diagnosis of squamous cell carcinoma of the tongue at clinical stage T3–T4.<br>Exclusion criteria: Patients younger than 18 or older than 75 years; were	Vehicle: albumin nanoparticles<br><br>Drug: polyoxyethylated oil free paclitaxel<br><br>Intraarterially injection at starting dose of 120 mg/m^2 was increased by 30 mg/m^2	Tumor response: physical examination and CT. PET was also performed. The response was classified as complete response (complete disappearance of all clinical and radiologic evidence of disease), partial response	No treatment	Tumor response: Eighteen patients (78%) had a clinical and radiologic objective response (complete, 26%; partial, 52%). Three patients (13%) showed stable disease, and two (9%) showed	A new polyoxyethylated castor oil and alcohol free formulation of the taxane paclitaxel has shown less systemic toxicity in preliminary clinical trials than commercially available formulations and is well tolerated locally even at

		pregnant or lactating; had undergone previous treatment; or had distant metastases.<br>Recruitment period: not stated<br>Number of participants: 23<br>Number analyzed: 23<br>Tumor site: tongue	at 3 subsequent levels each 4 weeks.	(≥ 50% decrease in tumor size), stable disease (< 50% decrease or < 25% increase in tumor size), or tumor progression (25% increase in tumor size).<br>Adverse effects: toxicity was assessed by a complete blood count, physical examination, ECG, and measurement of cutaneous toxicity. Toxicity was graded according to criteria set by the World Health Organization.<br>Duration of follow-up: 5-12 months		disease progression.<br><br>Adverse effects:<br>The toxicities encountered were hematologic (grade 3) in two patients (8.6%) and neurologic (grade 4) in two patients (reversible paralysis of the facial nerve, 8.6%).	high concentrations.<br><br>Evaluations of intraarterial treatment therefore are still empiric, being based on local toxicity, systemic toxicity, and evidence of antitumor activity, as shown by an objective response.
Strieth (2014) [17]	Prospective open nonrandomized dose-escalating Phases I/II clinical trial without a control group.<br><br>Level of evidence: IV	Inclusion criteria: age >18 years, life expectancy >4 months, and Karnofsky performance status<br>>60 points.<br><br>Exclusion criteria: secondary malignant disease, cerebral	Vehicle: liposomes of 1,2-dioleoyl-3-trimethylammonium-propane (DOTAP) and 1,2-dioleoyl-sn-glycero-3-phosphocholine.<br><br>Drug: purified paclitaxel	Tumor response: imaging studies with either contrast-enhanced CT or MRI.<br>A reduction of tumor volumes by 50% was defined as “partial response” and an increase by 25% as “progressive	Previously surgery and/or radiochemotherapy.	Tumor response: tumor volume measurements revealed stable disease in 4 of 5 cases. Reproducible dose-dependent blood flow reductions in skin metastases during ET infusions provide	Effective paclitaxel doses (0.55 mg/kg b.w. or 1.10 mg/kg b.w.) applied in ET liposomal formulations are far below the doses of conventional paclitaxel usually given in clinical practice (3–5 mg/kg b.w.), which may also be

		metastases, or were still recovering after primary tumor therapy, inflammatory disease. Unacceptable liver function.<br><br>Recruitment period: not stated<br>Number of participants: 07<br>Number analyzed: 05<br>Tumor site: hypopharynx	One group (n=3) received 3 infusions of ET at the lower dose of 0.55 mg paclitaxel/kg.<br>Another group (n=4, including two re-entries from the lower dose group) received 3 infusions of ET at the higher dose of 1.1 mg paclitaxel/kg	disease.” Changes in between were considered as “stable disease.”<br>Serological concentrations of the tumor markers Serpin B4, carcinoembryonic antigen (CEA), and cytokeratin 19 fragments (Cyfra 21-1) were analyzed before and after the infusions.<br><br>Adverse effects: toxicities were assessed using the NCI Common Terminology Criteria for Adverse Events.<br><br>Duration of follow-up: 2 weeks after the last infusion		evidence of biological effectiveness.<br><br>Adverse effects:<br>Only adverse events of grade 1 or 2 – in particular fatigue, chills, and hypertension occurred.	a reason for the favorable safety profile.
Caponigro (2000)<br>[3]	Phase I<br><br>Level of evidence: IV	Inclusion criteria: Patients with recurrent HNC after first line chemo/radiotherapy, or metastatic, head and neck cancer. Eastern	Vehicle: pegylated liposome<br><br>Drug: doxorubicin<br><br>The compound was	Tumor response: evaluated by physical examination, performance status recording, chest X-ray, cervical computed tomography scan (CT) or	Patients had previously undergone radiotherapy (10 patients), chemotherapy	Tumor response: One complete response (4%) and seven partial responses (29%) were observed, for an overall response rate of 33%	The overall response rate of 33% well matches that observable with the most active single agents in the same patient population.

		Cooperative Oncology Group performance status of 0-2, adequate baseline organ function, life expectancy of at least three months.<br><br>Exclusion criteria: Patients that received more than one line of prior chemotherapy or had completed prior antitumor treatment less than a month before inclusion.<br><br>Recruitment period: from July 1998 to September 1999<br>Number of participants: 24<br>Number analyzed: 24<br>Tumor site: oral cavity (10), oropharynx (4), nasopharynx (1), maxillary sinus (2) and larynx (7).	administered at the initial dose of 30 mg/m^2 and subsequently escalated by 5 mg/m^2 per step.	magnetic resonance imaging (MRI). Standard WHO criteria were used for response assessment.<br><br>Adverse effects: complete blood cell (CBC) count with differential, serum chemistries, urinanalysis and ECG.<br><br>Duration of follow-up: 13 months	(4 patients) or radio and chemotherapy (10 patients)	(95% CI: 16%-55%).<br><br>Adverse effects: three out of six patients had grade 3 stomatitis. Stomatitis occurred in 11 patients across all dose levels, considering all delivered cycles. Neutropenia occurred in 10 of 24 patients, but reached grade 4 in only 2 patients at fourth dose level. Skin toxicity, mainly appearing in the form of palmar-plantar erythrodysesestesia, was the most frequent toxicity, occurring in 14 patients.	
Rosenthal (2002) [2]	Phase I<br><br>Level of evidence: IV	Inclusion criteria: histologically proven, locoregionally advanced	Vehicle: ethoxypolyethylene glycol liposomes	Tumor response: annual x-rays and biopsy to confirm recurrence.	Concurrent with RT (60–72 Gy in 6–7 weeks).	Tumor response 3 patients had local recurrence or distant	The prolonged half-life of liposomal chemotherapy agents and

		HNC. ≤25% predicted 5-year survival or <50% predicted 5-year survival. Patients must have had a Karnofsky Performance Status of at least 60% and adequate bone marrow, liver, and renal function.<br><br>Exclusion criteria: pregnancy or lactating, nasopharyngeal carcinoma, pre-existing neuropathy, a history of allergic reaction to a platinum agent, significant bilateral hearing loss, or prior head and neck RT.<br><br>Recruitment period: not stated<br>Number of participants: 20<br>Number analyzed: 17<br>Tumor site: oropharynx (1), anterior tonsillar pillar/retromolar trigone (8), oral cavity (6),	Drug: cisplatin<br><br>Dose escalated from 20–200 mg/m^2 in six dose levels intravenously injected twice two weeks.	Complete response (CR), partial response (PR), stable disease, and progression were defined according to South west Oncology Group criteria. The survival rate was assessed by Kaplan-Meier method.<br><br>Adverse effects: Toxicities were graded by the National Cancer Institute Common Toxicity Criteria.<br><br>Duration of follow-up: 36 months		metastases and died of disease. Eight patients were without evidence of disease at last follow up. Nine patients died of disease progression<br>Estimated overall survival rate was 41% and disease-free survival was 25%.<br><br>Adverse effects: Two had reversible Grade 3 liver toxicity or rash. Three patients had a Grade 1, and one had a Grade 2 infusion reaction. Four patients had transiently elevated transaminases: Grade 1 (n = 1), Grade 2 (n = 1), and Grade 3 (n = 2). Grade 3 neutropenia occurred in one patient. There was no ototoxicity, neurotoxicity, or nephrotoxicity. In-field	their potential improved therapeutic indices as demonstrated by the relative paucity of severe toxicities even at the highest doses

		hypopharynx (1) and sinus/other (4)				radiation skin and mucosal toxicities did not appear to be intensified.	
Faivre (2004) [4]	Phase I-II<br><br>Level of evidence: IV	Inclusion criteria: patients with histologically-proven, locally recurrent or metastatic measurable CT scan or MRI) HNC and at least 3 weeks interval from last prior chemotherapy regimen, World Health Organisation (WHO) performance status 0–2 with a life expectancy of more than three months.<br><br>Exclusion criteria: history of cardiopathy with congestive heart failure, hypersensibility to anthracyclines or previous hypersensibility reaction to Cremophor-containing products and serious concomitant illness or medical condition.	Vehicle: Pegylated liposomes<br><br>Drug: Doxorubicin<br><br>Intravenous infusion over 1h at an initial dose of 35 mg/m^2, every 3 weeks. In the first phase of the study, 15 patients received a dose of 35 mg/m^2, every 3 weeks, the following 11 patients group were treated at 45 mg/m^2.	Tumor response: Response evaluation was carried out weekly with a clinical examination and imaging evaluation (CT scan and/or MRI) was performed every 2 cycles.<br><br>Adverse effects: Toxicity was evaluated after each cycle according to the National Cancer Institute Common Toxicity Criteria.<br><br>Duration of follow-up: at least 4 weeks.	Previous Therapy: Radiation therapy, Chemotherapy or both.	Tumor response: 4 patients presented objective responses (17%, 95% CI 0.5–32%). The antitumor activity was observed in patients with local recurrence in an irradiated area after 2 cycles, but no objective response was observed in patients with distant metastasis. 8 patients presented tumour stabilization as their best response. The median time to tumour progression and survival were 3.5 and 4.6 months, respectively. Among the 4 responding patients, 2 experienced necrosis of the bulk of the tumour.	Due to a high tumor tissue distribution of the drug tumour necrosis, ulceration and bleeding can be induced. Careful utilization of the drug is required for the treatment of tumours relapsing in irradiated areas.

		Recruitment period: not stated<br>Number of participants: 26<br>Number analyzed: 24<br>Tumor sites: Oropharynx (15), Oral cavity (5), Hypopharynx (4), Nasopharynx (1) and maxillary sinus (1).				Adverse effects: grade 3–4 neutropenia was observed in only 2 patients. There were no grade 3–4 mucosal, skin, digestive, cardiac or hepatic toxicities.	
Damascelli (2001)<br>[8]	Phase I<br><br>Level of evidence: IV	Inclusion criteria: Patients with histologic diagnosis of locally advanced squamous cell carcinoma of the head and neck with without previous treatment; Eastern Cooperative Oncology Group performance status of less than 2; previous chemotherapy, with exclusion of taxanes, completed at least 4 weeks before study nrollment; life expectancy longer than 3 months;<br>Exclusion criteria: Patients with formal	Vehicle: albumin nanoparticles<br><br>Drug: polyoxyethylated oil free paclitaxel<br><br>Administered percutaneous catheterization of the neck vessels<br>Three treatment cycles were planned, with a 4-week interval between cycles (in 2 patients 4 cycles were performed). The starting dose of 120 mg/m^2 was increased by 30 mg/m^2 at each subsequent	Tumor response: computed tomographic scans or magnetic resonance imaging were performed at baseline and before each treatment.<br><br>Adverse effects: All toxicities were graded according to World Health Organization (WHO) toxicity criteria. The MTD was defined as the dose level below that which induced a limiting toxicity in at least three of	Previously surgery, chemotherapy and/or radiotherapy	Tumor response: 3 patients with no previous treatment had complete responses. Nineteen partial responses were observed (6 previously treated patients and 13 not previously treated). The sum of complete and partial responses was 75.85% (complete response, 10.34%; partial response, 65.51 %). Six of the remaining seven assessable patients had received previous	This treatment do not required premedication, is easy and reproducible and has acceptable toxicity.

		contraindications or in whom transfemoral catheterization/angiography was not possible and those with severe cardiopathy were excluded.<br><br>Recruitment period: not stated<br>Number of participants: 31<br>Number analyzed: 29<br>Tumor sites: Tongue 10, Maxillary sinus 2, Floor of mouth 1, Soft tissues of the neck 5, Laryngopharynx 3, Overlapping lesion of oro/hypopharynx 1 Larynx 1 Piriform sinus 1, Retromolar trigone 2, Oropharynx 2, Overlapping lesion of<br>tonsil and palate 3	level. Each level consisted of a group of six cycles.	six cycles.<br><br>Duration of follow-up: 3–13 months		treatment, and of these one progressed, four had stable disease, and one developed a massive tumor necrosis. The last patient, not previously treated, showed stable disease.<br><br>Adverse effects: The dose-limiting toxicity was myelosuppression.	
Damascelli (2007) [5]	Phase II<br><br>Level of evidence: IV	Inclusion criteria: biopsy-proven SCC of the oral cavity, oropharynx, or hypopharynx (stage T3/4, any nodal stage).	Vehicle: albumin nanoparticles<br><br>Drug: paclitaxel	Tumor response: Clinical and radiological response were considered a complete response (CR) if there was no clinical or	Patients subsequently underwent the definitive treatment.	Tumor response: clinical response (15), partial response (30), stable disease (7) and progression disease (8).	A new class of macromolecular drugs make local administration more attractive as a means to

		Exclusion criteria: patients younger than 18 or greater than 75 years, pregnancy, previous cancer treatment of any kind, distant metastases, impaired renal or hepatic function.<br>Recruitment period: from May 2000 to January 2004<br>Number of participants: 60<br>Number analyzed: 60<br>Tumor sites: oral cavity 30, hypopharynx 3, Oropharynx 27	Two to four cycles of infusions into the external carotid artery or one of its branches, without premedication, at an initial dose of 230 mg/m2 and subsequently a reduced dose of 150 mg/m^2.	radiologic evidence of disease and a partial response (PR) if the tumor size had decreased by 50% or more. Stable disease was defined by a reduction in tumor size of less than 50% or an increase of less than 25% and no appearance of new lesions. Disease progression was defined as an increase in tumor size of 25% or more or appearance of new lesions.<br><br>Adverse effects: Toxicity was assessed according to World Health Organization criteria of six cycles.<br><br>Duration of follow-up: 3 weeks after de last infusion.		Adverse effects: High-grade bone marrow depression was rare. An unexpected toxicity was reversible facial nerve palsy on the side of infusion, which occurred in six patients at initial dosage. Reduction of the dose eliminated this specific toxicity without any loss of efficacy.	achieve rapid local control with low systemic toxicity before definitive treatment is undertaken. The heterogeneity of definitive treatment in our study makes it impossible to draw conclusions as to the impact of this treatment on survival. However, the results to date indicate that intraarterial administration of nanoparticle albumin–bound paclitaxel alone or in combination with other agents warrants further investigation.

Supplementary Table S2. Characteristics of the excluded studies after full text screening**.**

Study	Reason for exclusion
ABU-KHALAF 2015 [39]	Less than 50% of participants in trial have HNC
ADKINS 2013 [40]	Intervention concomitant with addition conventional chemotherapies
ANDO 2012 [41]	Less than 50% of participants in trial have HNC
CHANG 2015 [42]	Less than 50% of participants in trial have HNC
CHIANG 2016 [43]	Less than 50% of participants in trial have HNC
CHIEN 2009 [44]	Less than 50% of participants in trial have HNC
DEEKEN 2013 [45]	Less than 50% of participants in trial have HNC
DIAZ-PADILLA 2011 [46]	Abstract only, and no subsequent publication found March 19
JANINIS 2004 [47]	Intervention concomitant with addition conventional chemotherapies
KOVACS 2002 [48]	The treatment does not include nanoformulation
LEY 2017 [49]	Retrospective study
LI 2017 [50]	Intervention concomitant with addition conventional chemotherapies
LOONG 2014 [51]	Intervention concomitant with addition conventional chemotherapies
MAMOTO 2012 [41]	Less than 50% of participants in trial have HNC
MARKMAN 2016 [52]	Abstract only, and no subsequent publication found March 19
MEIQI 2018 [53]	Less than 50% of participants in trial have HNC
MITA 2007 [54]	Less than 50% of participants in trial have HNC
NYMAN 2005 [55]	Less than 50% of participants in trial have HNC
SEGAL 2019 [56]	The treatment does not include nanoformulation
SENZER 2013 [57]	Less than 50% of participants in trial have HNC
SOLOMON 2015 [58]	Less than 50% of participants in trial have HNC
STARODUB 2015	The treatment does not include nanoformulation and less than

[59]	50% of participants in trial have head and neck cancer
TEVAARWERK 2009 [60]	Less than 50% of participants in trial have HNC
TOURNEU 2017 [61]	Abstract only, and no subsequent publication found March 19
VELLECA 2010 [62]	The treatment does not include nanoformulation
VILLARET 2002 [63]	The treatment includes genetherapy
WEISS 2012 [64]	The treatment does not include nanoformulation
YOO 2001 [65]	Less than 50% of participants in trial have HNC
ZHANG 2009 [66]	Less than 50% of participants in trial have HNC
ZUKERMAN 2014 [67]	Less than 50% of participants in trial have HNC
